# Challenges and Opportunities in the Sustainable Improvement of Carrot Production

**DOI:** 10.3390/plants13152092

**Published:** 2024-07-28

**Authors:** Antonello Paparella, Prasada Rao Kongala, Annalisa Serio, Chiara Rossi, Liora Shaltiel-Harpaza, Amjad M. Husaini, Mwafaq Ibdah

**Affiliations:** 1 Department of Bioscience and Technology for Food, Agriculture and Environment, University of Teramo, Via R. Balzarini 1, 64100 Teramo, Italy; apaparella@unite.it (A.P.); aserio@unite.it (A.S.); crossi@unite.it (C.R.); 2 Newe Yaar Research Center, Agricultural Research Organization, Ramat Yishay 30095, Israel; 3 Migal Galilee Research Institute, P.O. Box 831, Kiryat Shmona 11016, Israel; liorat@migal.org.il; 4 Environmental Sciences Department, Faculty of Sciences and Technology, Tel Hai College, P.O. Box 831, Kiryat Shmona 11016, Israel; 5 Genome Engineering and Societal Biotechnology Lab, Division of Plant Biotechnology, SKUAST-K, Shalimar, Srinagar 19005, Jammu and Kashmir, India; amjadhusaini@skuastkashmir.ac.in

**Keywords:** abiotic stress, agriculture, biotic stress, carrot, production

## Abstract

From an agricultural perspective, carrots are a significant tap root vegetable crop in the *Apiaceae* family because of their nutritional value, health advantages, and economic importance. The edible part of a carrot, known as the storage root, contains various beneficial compounds, such as carotenoids, anthocyanins, dietary fiber, vitamins, and other nutrients. It has a crucial role in human nutrition as a significant vegetable and raw material in the nutraceutical, food, and pharmaceutical industries. The cultivation of carrot fields is susceptible to a wide range of biotic and abiotic hazards, which can significantly damage the plants’ health and decrease yield and quality. Scientific research mostly focuses on important biotic stressors, including pests, such as nematodes and carrot flies, as well as diseases, such as cavity spots, crown or cottony rot, black rot, and leaf blight, caused by bacteria, fungi, and oomycetes. The emerging challenges in the field include gaining a comprehensive understanding of the interaction between hosts and pathogens in the carrot–pathogen system, identifying the elements that contribute to disease development, expanding knowledge of systemic treatments, exploring host resistance mechanisms, developing integrated control programs, and enhancing resistance through breeding approaches. In fact, the primary carrot-growing regions in tropical and subtropical climates are experiencing abiotic pressures, such as drought, salinity, and heat stress, which limit carrot production. This review provides an extensive, up-to-date overview of the literature on biotic and abiotic factors for enhanced and sustainable carrot production, considering the use of different technologies for the shelf-life extension of carrots. Therefore, it addresses the current issues in the carrot production chain, opening new perspectives for the exploration of carrots both as a food commodity and as a source of natural compounds.

## 1. Introduction

Food security and sustainable agriculture are crucial global priorities for human civilization and sustainable development. Biotic and abiotic stresses, as well as climate change, threaten agricultural production. For this reason, the development of sustainable solutions for agricultural production is urgent for the safety of both the planet and humans. In this regard, the carrots of the *Apiaceae* family and their yields in production areas are linked to biotic and abiotic threats that limit crop potential and associated industry.

*Apiaceae* is a family of vegetables and medicinal plants that holds 434 genera and nearly 3780 species [1], including many vegetable crops that are rich in flavonoids, carotenoids, coumarin, coumarin derivatives, vitamins, and minerals [2]. All over the world, carrots are among the top 10 vegetables for agricultural production [3]. The worldwide production of carrots and turnips reached 40.24 Mt from 1,082,967 ha in 2020 and about 41.67 Mt from 1,096,007 ha in 2021, with enormous horticultural and economic importance (FAO STAT, https://www.fao.org/faostat/en/#data/QCL, accessed on 26 June 2024). China, Russia, and the United States account for the main part, 34%, of global production [4]. The *Apiaceae* family includes carrots (*Daucus carota* L.), which are the most important *Apiaceae* cultivated worldwide. They originated in Middle Asia near Afghanistan [5,6] and gradually expanded into the Mediterranean region [7]. The earliest carrots were mainly purple or yellow, with some white or black species, instead of orange [8].

**Figure 1:**
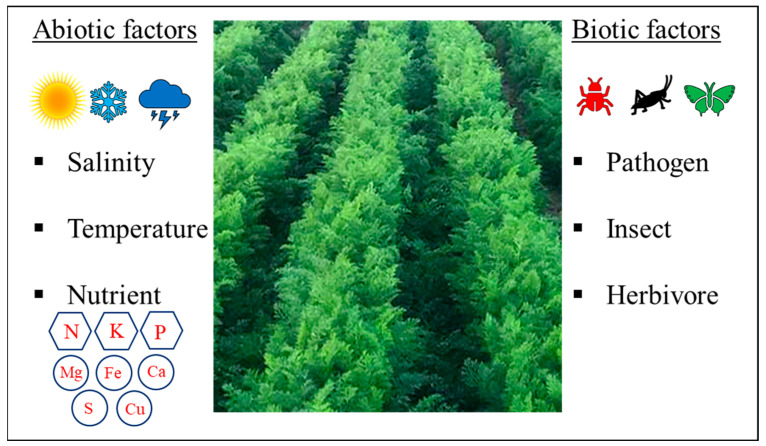
Photography of a carrot field depicting crops prone to abiotic and biotic factors affecting production and plant health (personal photos).

The cultivation of carrots involves various problems that arise at each stage of crop production and require attention and protection (Figure 1). These threats result in a reduction in crop yield and quality, which in turn leads to financial losses. Therefore, this review aims to provide a thorough analysis of the current scientific literature on carrot production, aiming to address challenges in the field and enhance the quality of carrots, offering new opportunities for utilizing carrots as a food commodity and as a source of bioactive compounds. This extensive analysis examines the wide range of biotic and abiotic stresses that can damage carrots, along with methods to control these factors. Moreover, different strategies to extend the shelf life of carrots during post-harvest are examined to promote improved and environmentally friendly production.

## 2. Bioactive Compounds Obtained from Carrots and Potential Applications

**Figure 2:**
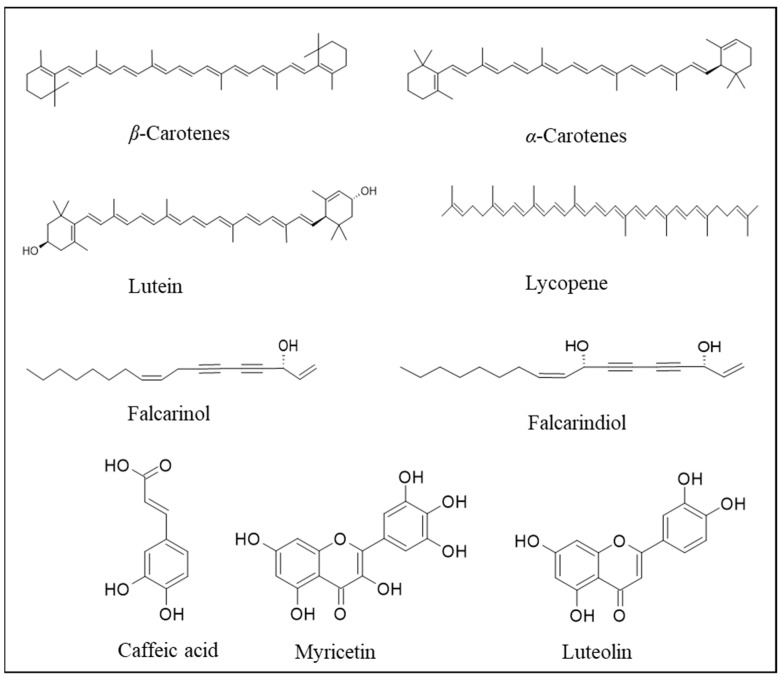
Carrot-root-sourced prominent nutraceuticals and other significant metabolites.

The species *D. carota* itself is a source of bioactive compounds that can be explored for different applications. The major phytochemicals in carrot roots are carotenoids (*α*- and *β*-carotenes, lutein, and lycopene), phenolic compounds (chlorogenic acid derivatives, caffeic acid, myricetin, luteolin, etc.), polyacetylenes (falcarinol and falcarindiol), and vitamins (vitamins C, E, K, B_1_, and B_4_; Figure 2), all considered as high-value bioactive molecules. Moreover, among fruits and vegetables, carrots are the richest source of *β*-carotene, which is the vitamin A precursor [9]. Carotenoids are great singlet-oxygen scavengers. Furthermore, diets rich in carotenoids, ascorbic acid, tocopherol, and non-vitamin antioxidants, such as anthocyanins and phenolic compounds, contribute to protecting DNA and proteins from oxidative processes [10]. Noticeably, black or purple carrots are particularly rich in acylated anthocyanins, which exert high antioxidant activity and nutraceutical features [11].

**Figure 3:**
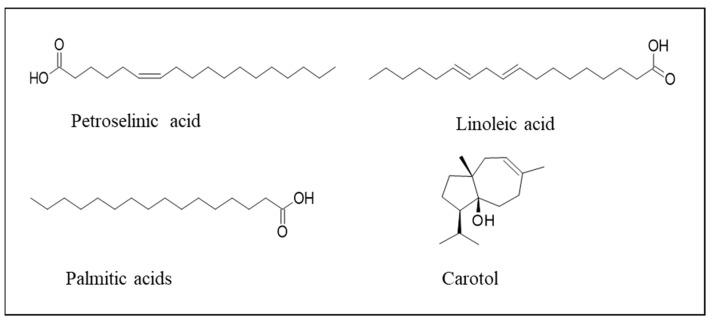
Major bioactive compounds obtained from carrot seed essential oil.

Carrot seeds are rich in petroselinic, linoleic, and palmitic acids, proteins, and fibers, and from them, both oil and essential oil can be extracted (Figure 3). Carotol is the main component of both seed edible oil (30.55%) and seed essential oil (66.78%) [12]. Traditional medicine suggests carrot seed EO as a tonic to treat digestive problems.

This EO also has analgesic, anti-inflammatory, antimicrobial, and antioxidant activity [13], and it is generally regarded as safe when ingested in low amounts as food flavoring. Carrot seed EO is the main source of carotol used in cosmetics for fragrance synthesis. Depending on the chemotype, this EO shows moderate and non-specific toxicity on different cell lines [13].

The EO can also be extracted from the dried fruit. Also, in this case, the most important compounds are sesquiterpenic molecules, such as *β*-caryophyllene, and the alcohols, carotol and daucol. Carrot EO exerts antimicrobial activity on bacteria and fungi, with a greater effect on Gram-positive bacteria, as it is usually observed for other EOs. Also, an extract obtained by supercritical fluid extraction (SFE) by means of carbon dioxide had carotol as the main compound (30.3%), and good quantities of geranyl acetate (7.22%), *β*-caryophyllene (6.47%), and daucol (2.46%).

**Figure 4:**
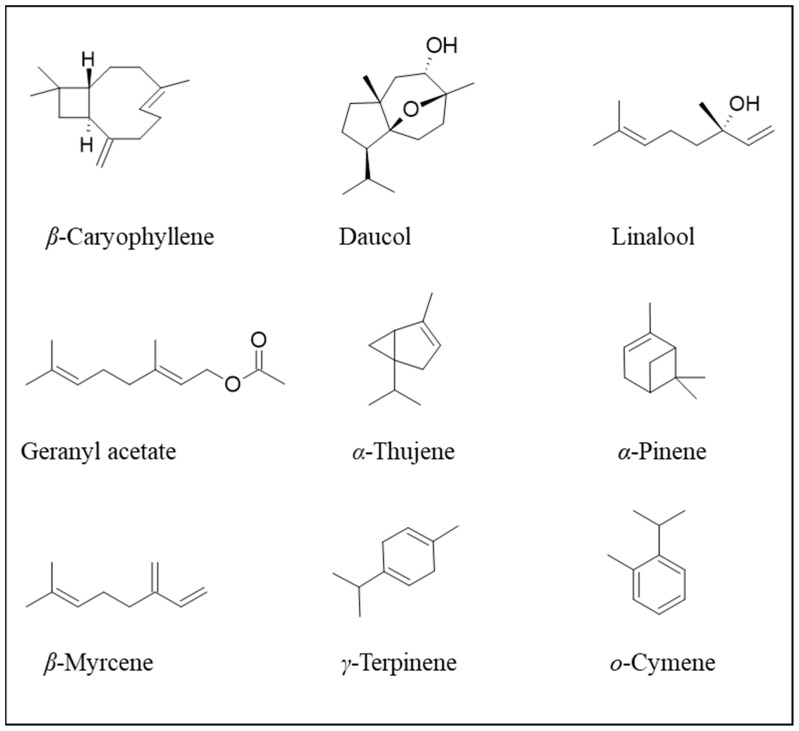
Major volatile organic compounds found in the carrot essential oil with different biological activities.

Still, it did not contain lighter components present in the EO, such as *α*-thujene, *α*-pinene, *β*-myrcene, *γ*-terpinene, *o*-cymene, and linalool (Figure 4). EO and SFE showed antimicrobial activity against Gram-positive bacteria, such as *Bacillus subtilis*, *B. cereus*, and *Rhodococcus equi* [14]. Carotol itself exerts fungicidal activity on *Alternaria alternata.*

An EO extracted from carrot umbels showed minimal inhibitory concentrations of 0.32–0.64 µL/mL against *Bacillus subtilis, Staphylococcus aureus*, and *Listeria monocytogenes,* and was also effective on dermatophyte strains and *Cryptococcus neoformans* (MIC of 0.16 µL/mL). In particular, the EO inhibited germ tube formation and filamentation (essential for virulence) in *Candida albicans* at very low concentrations, decreasing biofilm mass and cell viability [15]. As biofilm is difficult to prevent and eradicate, these results could be useful for implementing strategies to counteract candidiasis. The same EO demonstrated anti-inflammatory activity, decreasing the production of nitric oxide (NO), which is a mediator in the inflammatory response, in LPS-stimulated macrophages at concentrations safe for macrophages, hepatocytes, and epithelial cells [15].

Interestingly, a commercial wild carrot EO, rich in methyl isoeugenol (60.7%), and an EO extracted from umbels of wild carrots collected in Illinois had a toxic effect on mosquito larvae, such as *Aedes aegypti*, *Culex pipiens* L., and *Culex restuans* Theobald, therefore suggesting an application as a biopesticide [16]. In addition, a carrot seed EO, rich in carotol (>70% *w*/*w*), showed high repellency against *Aedes aegypti* and *Anopheles quadrimaculatus.* Even carotol alone showed a biting deterrent activity similar to diet in laboratory studies. Moreover, both carotol and carrot seed EO showed good repellency in skin application assays, showing the potential to be explored as a natural repellent in commercial formulations [17].

Moreover, different medical applications for EOs and solvent extracts have been proposed, and in detail, carrot bioactive compounds, such as lutein, beta-carotene, and polyacetylenes, as well as EO, were demonstrated to possess anticancer activity against different carcinoma and leukemia cell lines, ethanolic extracts rich in flavonoids and phenolic derivatives accelerate the wound healing process, while coumarin glycosides lower blood pressure and relax muscles. Carrot extract has been proven to have gastroprotective activity, thus supporting the traditional use in gastric ulcer and acidity treatment [18]. Carrot seed EO is used as a tonic and stimulant for skin problems and to treat hepatic and renal insufficiency [14]. On the other hand, it needs to be taken into account that bioactive polyacetylene compounds negatively impact the taste of carrot roots because they increase bitterness [19].

Interestingly, bioactive compounds are also contained in carrot waste and, therefore, food industries and research partners are focusing their activity on the recovery and valorization of these molecules from waste to enrich the nutritional profile of food products and beverages [10].

## 3. Biotic Threats: Carrot Diseases

The carrot crop is damaged or infected by over 150 species of insects, mites, nematodes, fungi, viruses, bacteria, or phytoplasmas. Among these, pests, such as nematodes, carrot flies, and diseases, including cavity spots, crown or cottony rot, black rot, or leaf blight, emerged as major challenges worldwide [20].

The interaction between carrots and pathogens, which leads to the development of disease, is influenced by various factors, including agroecological parameters, host specificity, growing stage, phytotoxic compounds, and the virulence of the strain. A comprehensive comprehension of disease progression, together with the analysis of genetic and observable traits, will facilitate the identification and cultivation of superior carrot cultivars. The management tactics are always changing based on the specific characteristics of the carrot–pathogen interaction and the roles of the geographical agro-climate system in crop production.

### 3.1. Bacterial Diseases

#### 3.1.1. Bacterial Leaf Blight

##### *Xanthomonas hortorum* pv. *carotae* 

**Figure 5:**
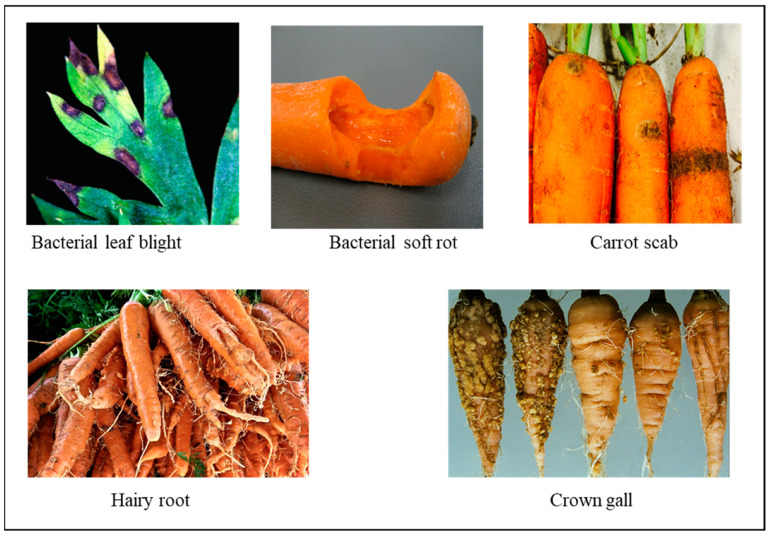
Pictures of carrot leaves and roots depicting bacterial diseases and symptoms (personal photos).

*Xanthomonas hortorum* pv. *carotae* (*Xhc*) causes carrot bacterial leaf blight, which can be spread through seeds [21]. Carrot fields throughout Europe, North America, and Asia frequently show signs of its presence [22]. According to Pruvost et al. [23], the seed serves as a primary source of inoculum. The bacteria can persist in carrot remnants and can be transmitted through carrot seeds, but they are unable to thrive in the soil in the absence of debris. Temperatures ranging from 25 to 30 °C (77° to 86 °F) are conducive to the spread of infection and the development of diseases (Figure 5). The bacteria are dispersed through the action of water splashing, and plant-to-plant dispersal can occur under times of heavy dew. Control measures for bacterial blight are often unnecessary in the majority of regions where carrots are grown. To achieve optimal disease growth, either rain or spray watering is required.

Within carrot fields, these brown spots are frequently identified as the initial signs of the disease, which are then followed by small, irregular yellow lesions on the leaves, stems, and petioles. These lesions may resemble water-soaked necrotic lesions [24]. The leaf exhibits irregular brown patches, typically originating from the edges of the leaf. Lesions first exhibit an asymmetrical golden halo and may appear saturated with water. Spots merge together and result in leaf disease, while leaf petioles develop dark brown streaks. Floral components might also experience blight.

An adhesive, amber-colored bacterial secretion (a diagnostic sign of the disease) could be seen on foliage or trickling down on leaf stems and flower stalks. The acceptable methods of control include: (i) cultural controls, (ii) the use of *Xanthomonas*-indexed seed or treated seed in hot water dips, (iii) the application of certain copper sulfate formulations through spraying, (iv) furrow or drip irrigation instead of sprinklers, (v) burying leftover carrot scraps to accelerate the process of decay, and (vi) preventing the persistent cultivation of carrots by implementing a crop rotation plan that spans two to three years.

#### 3.1.2. Bacterial Soft Rot

*Klebsiella variicola*

*Pectobacterium* spp.

*Dickeya dadantii*

Bacterial soft rot by *Klebsiella variicola* is a major constraint in carrot farms. It is one of the most devastating diseases [25]. Most bacterial soft rot agents are members of *Pectobacterium* spp. and *Dickeya,* with the former genus encompassing a constantly growing number of species varying in geographic distribution and host of isolation [26]. Carrots with severe soft rot symptoms on the tap root (Figure 5) have a putrid smell, wilting, and foliage collapse [25]. Biological agents, specifically strains A6 and P42 of *Bacillus velezensis*, have been found to be effective in managing soft rot disease in carrots. These strains have shown antagonistic properties against *K. variicola*, making them suitable biocontrol agents. This approach is considered more environmentally sustainable compared to the use of agrochemicals.

#### 3.1.3. Hairy Root

##### *Agrobacterium rhizogenes* 

Hairy root production in carrots is caused by the infection of *Agrobacterium rhizogenes*, resulting in the development of proliferative multi-branched adventitious roots at the site of infection [27]. Carrots grown in soils that are moderately dense and contain a high amount of decomposed organic matter often exhibit an overabundance of leaves (Figure 5) and generate roots that are hairy and forked. Additionally, the outer texture of these carrots tends to be rougher and coarser. Hairy roots serve as a biological platform for synthesizing a wide range of complex biomolecules.

#### 3.1.4. Crown Gall

##### *Agrobacterium tumefaciens* 

In carrots, crown gall is caused by *Agrobacterium tumefaciens* (Sm. and Town.), which can produce galls as a result of residing in a tumor-inducing (Ti) plasmid. The carrot crown gall manifests as tubular to irregular, yellow to tan galls located on the stem in close proximity to the crown or on the roots. Galls typically form at the junction of lateral roots and the tap root. Nevertheless, galls can form in any location where the plant has sustained damage (Figure 5). Multiple galls, varying in size, can develop on a plant during midsummer and continue growing in quantity and size until harvest. To address this issue, implementing cultural methods, such as employing extended crop rotations with onion, maize, oats, grasses, and other resistant crops, can potentially lead to a decrease in soil bacterial populations. Biological management of crown gall on stone fruits and roses has been successfully accomplished by introducing a harmless strain of *Agrobacterium radiobacter* (Beij. & Van Delden) Conn into these plants. This method has been documented by several studies [28,29,30].

#### 3.1.5. Scab

##### *Streptomyces scabiei* 

Scab disease of carrots is caused by three different bacteria: *Streptomyces acidiscabies*, *S. caviscabies*, and *S. scabiei*. These bacterial diseases affect the marketable production of carrots in both field and greenhouse settings [31,32,33]. Only a small number of *S. scabies* infections result in damping-off. Plants affected by scabies exhibit characteristic scab symptoms on their roots, where scab lesions are generated due to the aberrant growth of the host cells. This leads to the formation of corky tissue that is typically darker than healthy tissue (Figure 5). Lesions can occasionally be either depressed below or elevated above the level of the intact skin. Multiple individual lesions have the potential to merge together, resulting in the formation of contiguous scabby regions.

The severity of the scab is rarely significant enough to necessitate particular control techniques. Nevertheless, it is worth noting that alkaline soils tend to promote the growth of scab in other crops, including potatoes. Therefore, to mitigate the disease, it is advisable to refrain from cultivating carrots in alkaline soils or to use fertilizers that have an acidic effect, such as ammonium sulfate or sulfur, to decrease the pH of the soil. Cultivating carrots in soils that have a high capacity to retain moisture or implementing irrigation practices to ensure a consistent water supply can potentially decrease the occurrence of scab disease. Moreover, it is advisable for farmers to refrain from cultivating carrots on fields that have been previously used for potato farming. Implementing extended crop rotations involving small grains, grasses, or maize can potentially decrease the severity of scabs [31,34,35]. Thaxtomin A, a phytotoxin produced by *Streptomyces* spp., is the primary virulence determinant of scab in carrots. Due to the shared characteristics between potatoes and tap root crops in terms of infecting strains and the key virulence factor, thaxtomin A, it is advisable to evaluate the effectiveness of management methods developed for potatoes in controlling *Streptomyces* scab disease in carrots [36].

### 3.2. Fungal and Oomycete Diseases

#### 3.2.1. Alternaria Leaf Blights (ALB)

##### *Alternaria dauci* (J. G. Kühn)

**Figure 6:**
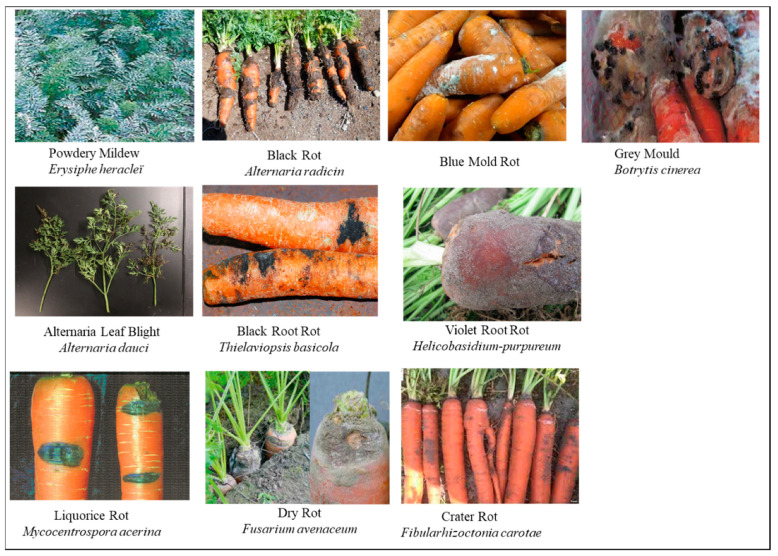
Photographs of carrot leaves and roots illustrating fungal and oomycete diseases and symptoms (personal photos).

Alternaria leaf blight (ALB) of carrots, caused by the fungus *A. dauci* [2], is one of the most common and destructive diseases worldwide [37]. Typically, the *A. dauci* infection leads to extensive deterioration of the leaves and main root, causing substantial reductions in crop productivity [38]. In the beginning, the infection affects the foliage and petioles, with small areas of dead tissue with varying forms and sizes (Figure 6) [33,39]. The spots gradually expand and merge until the entire leaf withers. Hence, the process of mechanical harvesting becomes challenging, ultimately resulting in a substantial decrease in crop production [5,6]. During epidemics, crop output reduction can reach 90% [40,41].

Investigating the resistance mechanisms of carrots to the leaf metabolites produced by *A. dauci* could be a promising avenue of research. The major leaf compounds found against *A. dauci* of carrot varieties and accessions were terpenes, such as myrcene, sabinene, *trans*-*α*-ocimene, limonene, germacrene D, *trans*-*α*-caryophyllene, *β*-caryophyllene, *β*-myrcene, and α-pinene [4,42,43].

The elucidation of the biosynthetic pathway for luteolin and apigenin derivatives, which are flavonoids, will provide a crucial foundation for conducting functional and genetic studies of flavone production in carrots. A study conducted by Koutouan et al. [44] demonstrated that the growth of *A. dauci* conidia was hindered by two secondary metabolites found in carrots, namely, falcarindiol and 6-methoxymellein. The cultivars that are resistant and susceptible to *A. dauci* showed varying levels of accumulation of falcarindiol in their leaves, suggesting that falcarindiol plays a role in resistance to *A. dauci* [45]. Currently, ALB stands as the most detrimental foliar disease. No known resistance gene can effectively combat this fungus [39]. At present, all resistant cultivars only possess partial resistance, thus requiring the continued use of fungicide treatments.

#### 3.2.2. Black Root Rot (Black Mold)

##### *Trichocladium basicola* (Berk and Broome)

Black root rot is a highly destructive disease that affects fresh carrots growing in muck soils after they have been harvested. Lesions consistently develop in the locations where wounds are acquired during the processes of harvesting, grading, and sorting. The symptoms consist of superficial black lesions that are irregularly and randomly distributed (Figure 6). These lesions range in size from 3 to 20 mm. These formations occur under high relative humidity conditions on roots that have been washed, sorted, and stored in polyethylene bags at temperatures exceeding 25 °C. Only the epidermis is affected by root discoloration caused by widespread fungal sporulation.

The disease occurrence is linked to the storage of recently harvested carrot roots at elevated temperatures and relative humidity. Storing carrots at the ideal temperature of 0 to 1 °C and relative humidity of 98 to 100% rarely poses a significant issue. Before grading, it is advisable to eliminate as much soil that is clinging to the roots as feasible. Moreover, refrigerating just-gathered carrots is recommended. The storage temperature must be kept below 7 °C. Carrots should be subjected to chemical control by immersing them in chlorinated water prior to being packed in plastic bags [46,47,48].

#### 3.2.3. Black Rot

##### *Alternaria radicina* 

Black rot is caused by the infection of *Alternaria radicina*, leading to the formation of black spots. The markings on the leaves and leaf stem are similar to those caused by *A. dauci*, characterized by irregular black lesions, especially along the margins of mature leaves. The infection can infiltrate the vascular bundle on the petioles, resulting in the leaf undergoing a yellowing process, wilting, and ultimately perishing (Figure 6). The size and pattern of the spots can range from tiny linear lines to very large round patches. Infections induced by *A. radicina* are generally less severe compared to those caused by *A*. *dauci*. *A. radicina* grows within a temperature range of −0.5 to +34 °C, specifically when the moisture level of the air reaches 92%. The disease primarily affects carrots during storage [39,49]. Infected carrots can also contaminate any nearby healthy carrots, serving as a source of infection.

*A. radicina* can be found on several parts of the plants, including seeds, umbels, foliage, petioles, and roots. On seedlings, it causes seed decay, damping-off, blackened hypocotyls, and malformed roots. On seeds, it forms a diffuse black weft of mycelium, which can cover the seeds and include black conidia. Additionally, the bottom portion of the tap root is destroyed. Seed-borne infection or planting in infested soil may lead to pre- and post-emergence damping-off. Affected seedlings have tan-brown to black lesions constricting the stem, which may be continuous. This lesion can grow from the soil level upwards and sometimes reach the cotyledons. Growers must implement at least eight years of crop rotation with crops other than carrot, dill, parsley, parsnip, and celery, using only seeds treated with hot water or a fungicide. In addition, crop debris should be removed immediately. For optimal preservation, it is recommended to maintain a storage temperature of approximately 0 °C and a humidity level of around 92% to minimize deterioration. The use of fungicides for chemical management can effectively decrease the occurrence of storage decay during the foliar phase [50].

#### 3.2.4. Crown Rot (Rhizoctonia Canker)

##### *Rhizoctonia solani* Kühn, Anamorph of *Thanatephorus cucumeris* 

Crown rot by *Rhizoctonia solani* also causes damping-off of carrot seedlings, usually more damaging on the roots of larger carrots, which results in a significant decrease in crop productivity [51,52,53]. The fungus has an extensive host range among vegetable crops [51,52,54]. Prolonged mid-season infections cause rot during storage. The earliest marks of crown rot are horizontal dark-brown lesions that form at the locations where lateral roots emerge from the tap root. These lesions may extend a few millimeters into the tap root, differently from cavity spot lesions caused by *Pythium* spp. The lesions of crown rot are numerous on the upper portion of the root. The disease is also characterized by the presence of a dark brown, dry rot that forms a band around the crown. The external foliage of the impacted roots withers and perishes, resulting in the plant having a limited number of larger internal leaves that remain erect [51,52,53]. Upon extracting the diseased roots, substantial amounts of dirt and mycelium can be found.

Carrot crown rot is a significant disease that severely limits or decreases the number of marketable carrots and overall profitability. The carrot crown displays several symptoms that impact its marketability, including ring crown rot, smooth crown rot, corky crown rot, soft watery crown rot, and black ring (Figure 6). Carrots that are infected will exhibit lesions on their roots, which can harm the overall health of the crop and result in lower grades for the saleable produce. With the exception of black rings, all other faults in carrots are considered significant and will result in the carrots being discarded as trash. On the other hand, carrots with black rings on the tops are classified as lower-grade and are sold for less than half the price of premium-grade carrots.

The most severe occurrence of crown rot and damping-off is observed at temperatures ranging from 20 to 28 °C, while infection or disease development is minimal below 16 °C. Optimal disease development occurs when soil moisture levels are above field capacity, which is about −0.1 bar [51,52]. The diverse soil factors and field conditions seem to have a huge influence on the types of crown rot symptoms, including (i) soil compaction caused by prolonged wet conditions and soil crusting, which appears to be a major contributing factor to crown rots, (ii) early rubbing friction in dry soil crust and other physical injuries that precede the development of ring crown rots, and (iii) infections in tall, dense crop canopy, such as *Sclerotinia* and other foliar diseases, causing soft watery crown rot in cool, wet conditions.

Carrot plants of all ages are equally vulnerable to *R. solani*, with crown rot being more severe in older plants. To decrease the occurrence of damping-off in carrot seeds, it is recommended to apply a fungicide to the seeds, as suggested in [53,55,56,57]. Moreover, the risk of crown rot should be reduced by treating the soil surface after cultivation to break up any crust that has formed on the top layer. In addition, carrot tops are encased in soil to shield them from drastic changes in moisture and temperature levels on the surface of the soil. Finally, trimming carrot tops either horizontally or vertically may potentially help reduce the occurrence of soft, watery, and black ring crown rot in carrots.

#### 3.2.5. Ring Rot Disease (Pythium Root Dieback)

*Pythium coloratum* (Vaartaja)

*Pythium irregulare* (Buisman)

*Pythium sulcatum* (Pratt & Mitchell)

*Pythium sylvaticum* (W.A. Campbell & J.W. Hendrix)

*Pythium ultimum* (Trow)

*Pythium sulcatum*, a soil-borne pathogenic oomycete that morphologically resembles a fungus, is responsible for the highly damaging cavity spot disease [7,58]. The characteristic symptoms consist of concave, round-to-elliptical lesions 2 to 5 mm-long [59]. The tap root may be branched and surrounded by several elongated lateral roots. In other cases, the size may be greater, but the growth is stunted or divided into multiple branches. The foliage often appears robust, although occasionally, it may appear stunted or wilted. Severely affected seedlings may wilt and die [58]. Mature plants have the potential to recuperate by developing a large number of side roots, but these plants usually yield tap roots of inferior quality. The disease has also been referred to as rusty root, lateral root dieback, and forked root [7,58].

The hyphae of *Pythium* spp. are hyaline and aseptate, except for old hyphae. Septa are found at the base of reproductive structures. Young hyphae exhibit cytoplasmic streaming, as observed by Van der Plaats-Niterink [60,61]. According to Howard et al. [62], carrot plants that were cultivated in sand contaminated with *P. ultimum* and kept at a soil moisture potential of −2.5 kPa exhibited a higher number of forked roots at a temperature of 23 °C, compared to 27 °C. *P. ultimum*, *P. aphanidermatum*, and *P. irregular* exhibit increased lethality toward carrot seedlings when exposed to a temperature of 35 °C instead of 25 °C.

The implementation of cultural techniques, including the cultivation of carrots on raised beds, has been found to effectively decrease the occurrence of root forking and improve the percentage of marketable carrots. Carrots should not be cultivated in fields with inadequate drainage or susceptible to flooding. Additionally, it has been demonstrated that precision seeding effectively decreases the occurrence of root dieback. Implementing crop rotations with cabbage, corn, mint, onion, and potato has the potential to decrease the occurrence of *Pythium* root dieback in subsequent carrot crops. Finally, commercial cultivars should possess a high level of tolerance to *Pythium* root dieback [63].

#### 3.2.6. Cavity Spot

*Pythium intermedium* (de Bary)

*Pythium irregulare* (Buisman)

*Pythium sulcatum* (Pratt & Mitchell)

*Pythium sylvaticum* (W.A. Campbell & J.W. Hendrix)

*Pythium ultimum* (Trow)

*Pythium violae* (Chesters & C.J. Hickman)

Cavity spots are caused by various *Pythium* species, including *P. violae*. Carrots infected by *Pythium* spp. show symptoms of root dieback and have numerous rusty-brown lateral roots [64]. Carrots planted in recently cleared land or cultivated fields where umbelliferous crops have never been grown may develop severe cavity spots.

Conversely, fields where carrots have been cultivated repeatedly may have no history of cavity spots. Fields known to produce carrots infected with cavity spots may not show disease from one year to the next, depending on environmental conditions. First, symptoms appear under intact periderm as sunken areas that are either gray or not discolored [65]. The cavities resemble elliptical lesions that are sunken a few millimeters below the root surface. The lesions are elongated horizontally, arranged randomly, and darkened with age.

Lesions vary in size, and secondary organisms may infect the carrot, causing rapid rotting. The size of the cavities expands proportionally with the growth of the roots. Vertical cracks are sometimes associated with the cavities. Regarding cultural methods, carrots on raised beds are used to reduce the likelihood of excessive soil moisture levels and avoid using fields with a history of cavity spots. The utilization of resistant cultivars will facilitate progress. The severity of cavity spots has been linked to the use of high rates of chemical fertilizers and to increases in soil moisture either early in the season or throughout maturation. Simultaneously, decreases are found in soils with a pH higher than 8 [66]. Carrots with cavities are not suitable for sale in their fresh state or for processing, and their overall yield can be significantly diminished.

### 3.3. Insects

#### 3.3.1. Carrot Psyllids

##### *Candidatus Liberibacter solanacearum* 

Carrot psyllids (*Trioza apicalis*, *Trioza anthrisci*, *Bactericera nigricornis*, and *B. trigonica*) are the insect vectors that feed on the carrot leaves, causing substantial damage to the growing crop. Moreover, the psyllids can transmit a bacterial pathogen called *Ca. L. solanacearum*, a vector-transmitted yet-unculturable alpha-proteobacterium associated with carrot diseases [67].

**Figure 7:**
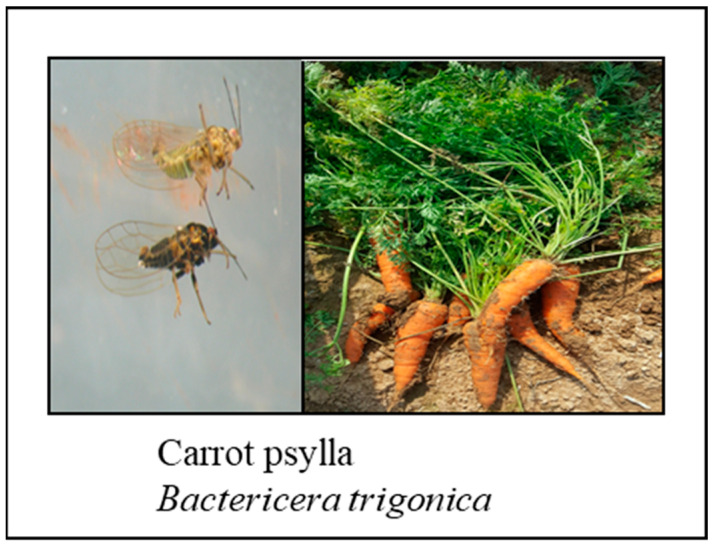
Photographs of carrot psylla and carrot symptoms (personal photos).

*Ca. L. solanacearum* has ten divergent haplotypes identified, which cause different diseases in host plants over a wide geographic distribution. The haplotypes C, D, and E cause diseases in carrots and celery in Europe [68]. The haplotype C is transmitted by *T. apicalis* Forster in northern Europe [69], whereas the haplotypes D and E are transmitted by *B. trigonica* Hodkinson in the Mediterranean area on both carrot and celery. The recently identified haplotype H was found to infect carrots and parsnips (Figure 7).

Carrot psyllid *T. apicalis* feeding exhibits typical symptoms of leaf curling and stunted growth of the shoot and root, whereas the symptoms associated with phloem limited pathogen *Ca. L. solanacearum* haplotype C bacterial infection, which causes leaf discoloration, and reduced the storage root weight [69]. Leaf curling is a rapid response to psyllid, but the *Ca. L. solanacearum* symptoms develop slowly and become visible 1.5 months after inoculation when the bacterial titer is high [69,70].

The shoot proliferation symptoms (i.e., witches’ broom) in carrots are caused by *Candidatus* Phytoplasma and *Spiroplasma* infection. The *Ca. L. solanacearum* haplotype-D-associated shoot proliferation symptoms are influenced by temperature, plant age, and vector load, being very sensitive at 30 °C and favored at 18 °C [71].

### 3.4. Microbial Ecology of Carrots

Vegetables, including carrots, can be contaminated by both spoiling and pathogenic microorganisms directly via the seeds or during cultivation, harvesting, post-harvesting procedures, processing, and storage, up to the distribution [72]. In particular, the microbial ecology of carrots is strictly related to the quality of the soil where they are cultivated. Moreover, raw or improperly composted manure, as well as low-quality water used for irrigation, may be an important source of microorganisms, including pathogens and antibiotic-resistant bacteria. Among the bacteria, Dharmarha et al. [73] reported the presence of *Gammaproteobacteria, Bacilli, Betaproteobacteria, Actinobacteria,* and *Alphaproteobacteria,* from the most to the least abundant, for a total of 114 different families, with 78% of bacteria belonging to the families *Pseudomonadaceae, Enterobacteriaceae, Oxalobacteriaceae*, *Bacillaceae*, and *Paenibacillaceae*. Although these families are common on other vegetables, it has to be underlined that *Enterobacteriaceae* also include pathogenic bacteria. For example, the presence of *Yersinia pseudotuberculosis* was associated with gastrointestinal disease in 2004 and 2006 in Finland [74,75]. Also, *Salmonella* spp. has been related to different vegetables, including carrots [76]. As regards non-pathogenic bacteria, the presence of psychotropic *Pseudomonas* spp., coliforms, and *Enterobacter* spp. is reported on fresh carrots and normally increases during refrigerated storage [72]. The total aerobic count can be as high as 7.9 Log CFU/g on whole carrots [72]. Also, *Dickeya, Pectobacterium* (both previously belonging to the genus *Erwinia*), and *Pseudomonas* are commonly reported in carrots, although not all *Pseudomonas* strains are responsible for spoilage. In addition, yeasts and molds can be recovered on carrots, deriving from in-field contamination, and their counts often arise during storage.

#### Spoilage

Carrots are among the most consumed vegetables worldwide—they are cultivated root vegetables, often stored for long times for year-round supplies, as in Northern Europe. Nevertheless, long-term storage impairs the nutritional and microbiological quality of the product [77]. Carrots contain about 90% of water, with 7.6% available carbohydrates and deficient amounts of lipids (0.2%) and proteins (1.1%) [78]. Considering the low fat and protein content, it can be inferred that much water is available for microbial development. Additionally, polysaccharides are converted into simple sugars, easily employable by microorganisms to sustain their growth during storage.

Carrots are exposed to colonization by fungi and bacteria already during their cultivation. In particular, when soil conditions are wet, spoilage is favored [79]. The same microorganisms can also be recovered from the fresh product. Microorganisms first grow on the surface of the vegetable, but some of them possess lytic enzymes, such as pectolytic and cellulolytic enzymes, allowing them entrance into inner tissues. The process is facilitated with fresh-cut carrots. The most common changes due to microbial growth are weight loss, bitterness, bacterial deterioration, and sprouting. Moreover, carrots quickly lose firmness while off-odors develop as a consequence of the high respiration rate and microbial growth [80]. The most common spoilage bacteria occurring on fresh, unprocessed carrots are those belonging to the genera *Dickeya*, *Pectobacterium*, and *Pseudomonas.* The first two are able to colonize carrots first in the field, where they can cause plant disease, and then post-harvest during storage, while *Pseudomonas* is mainly responsible for post-harvest spoilage. *P. viridiflava, fluorescens, cichorii*, and *marginalis*, as well as *P. carotovorum* subsp. *carotovorum* and *D. chrisanthemi* cause carrots to soft rot [77]. Particularly, *P. carotovorum* subsp. *carotovorum* can cause significant losses if left uncontrolled. Spoilage generally starts from the crown or root tip and continues rapidly toward the innermost region [81]. Apparently, the peel and the color of the carrot remain intact, while the root becomes watery, slimy, and soft, with a rotten odor. Moreover, secondary fungi often grow in rotten areas. Also*, Erwinia rhapontici* has been reported to cause carrot spoilage and is associated with cavity formation [77]. As already described, different fungi are related to plant diseases in the field. Nevertheless, some are also responsible for spoilage during storage in refrigerated or room-temperature conditions. In detail, *Botrytis cinerea* causes black lesions, where the production of grey spores can be noticed. When carrots are stored at room temperature, black root rot can occur. Moreover, *Chalaropsis thielavioides* and *Thielaviopsis basicola* are responsible for the black spots on the surface of the carrot, which can be covered entirely in a few days, making the vegetable unsuitable for consumption. Washing and refrigerated storage can help control microbial growth. Based on post-harvest storage methods, unprocessed carrots’ shelf life can vary from one week to one year or more.

## 4. Influence of Abiotic Stresses on Carrot

In vegetable crops, growth, development, and yield are affected by abiotic stresses, such as soil salinization, low and high temperatures, and drought. To overcome stresses and survive, crop plants evolve different protective mechanisms [82]. Crop plant improvement for high yield and tolerance to abiotic stresses by breeding are effective strategies, leading to sustainable agricultural production and safeguarding food supplies [83]. Investigating the physiological mechanisms and their regulation is important for the development of stress-tolerant plants using either conventional systems or bio-technological approaches [84,85]. Carrots are categorized as an excellent crop since they do not demand the warmer conditions required to produce vegetables, as tomatoes or cucumbers do. An optimal growing temperature of 17 °C with a range from 7 to 24 °C is typical [86] for carrot production in temperate climates. However, relatively little has been reported for high-temperature effects on carrot growth.

Abiotic stresses influence the changes in phenolic compounds in carrots. Oliveira et al. [87] observed that the activity of the phenylalanine ammonia-lyase (PAL) significantly increased with subsequent increments of 1000–1500% of total phenolic content after 72 h at 15 °C in wounding and moderate UV-C pretreatment. Hyperoxia storage even improved total phenolic increments by up to 2000%, partly profited by mild water stress. UV-C pretreatment has reduced PAL activity, favored by a higher electrolyte leakage. Post-harvest abiotic stresses resulting in phenolic accumulation of carrots leading to greater assimilation of antioxidant compounds can be used to increase the health-promoting properties of carrots, at the same time meeting food safety requirements related to the use of a moderate UV-C dose.

### 4.1. Salinity Stress

Carrots, as a glycophyte root crop, exhibit sensitivity to salinity [88]. They conveniently grow in soils containing low-sodium salts. The carrot accessions exhibit varied responses to salinity, and highly saline-growing carrots are also reported [89,90]. Under salt stress, carrot plants’ responses are inhibition of growth, abnormalities in morphological characters, and accumulation of malondialdehydes (MDA) membrane lipid degradation products. Biochemical activity reveals reduced soluble protein content and lower superoxide dismutase (SOD), catalase (CAT), and peroxidase (POD) activity. Tolerance under increased salt levels has been attributed to several mechanisms that enable plant growth and development.

Kamińska et al. [88] investigated the protective mechanisms against osmotic and ionic stresses involved in the salt tolerance of carrots. In this study, the salinity EC 3.15 dS m^−1^ was maintained for the doubled-haploid DH1 line (sensitive to salinity) and DLBA (exhibiting tolerance to salinity), a local variety (Fars region in Iran), to determine the changes in biochemical traits. It was observed that the tolerant DLBA variety was moderately determined constitutively. Even the exposure to saline soil caused a physiological response, more evident in the root. Thus, carrot plants adapted to stress conditions by osmotic adjustments and activation of the antioxidant system.

It was evident that osmoprotective proline and low molecular antioxidants, such as glutathione and ascorbic acid contents, were increased, with a decreased ratio of reduced to oxidized glutathione forms. All in all, these alterations indicate an effective ascorbate–glutathione cycle operation with a high activity of antioxidative enzymes, such as peroxidases, involved in resistance against extreme reactive oxygen species.

Kwolek et al. [91] studied carrot F2 lines segregated in salt tolerance levels, derived from cross-fertilization of two lines, one resistant (DLB-A, an Iranian line) and the other susceptible (2874B, a Polish breeding line) to salinity. At 150 mM NaCl, the early response of seedlings in the germination assay indicated that salinity stress increased the time required for germination up to 4 weeks from 1 to 2 weeks, and only 20% of seedlings were grown. All salt-stressed seedlings exhibited relatively normal morphology, besides the thickening of hypocotyls, roots, and cotyledons, with a chlorotic green-yellow coloration of all organs observed. Moreover, the increased water uptake could be a vital factor in carrot tolerance to salinity. Using genotyping-by-sequencing (GBS), Kwolek et al. [91] identified the regions in the genome of the carrots that were involved in tolerance to salinity, which accounted for the lethality of F2 plants sensitive to salinity. It was based on the expected deviation from the Mendelian segregation in the group of plants under stress, whereas no deviations were expected in controls. It was revealed that the incidence of SNP alleles in the F2 plants under stress differed compared to the control plants. Most polymorphisms exhibited partial segregation on chromosome 2 in the salt-treated lines but not in the control. Moreover, in the salt-treated F2 sub-population, only one variant of chromosome 2, heredity from the tolerant parent, was conserved. It likely bears dominant gene(s) acclimatizing resistance to salinity stress.

An experiment on carrots by Simpson et al. [92] revealed salt stress and ABA (abscisic acid)-induced expression of DcPSY2 (phytoene synthase (PSY) promoter fragment) by binding of AREB transcription factors (probably DcAREB3) to the ABREs noticed in the promoter of DcPSY2. In the transcriptome of the carrot, three ABRE-binding protein (DcAREB) transcription factor candidates, localized in the nucleus, were identified. However, only one of the three, namely, DcAREB3, was induced under ABA treatment in carrot roots. AREB transcription factors were discovered in the carrot DcPSY2 promoter and expressed reporter genes by transactivation. Furthermore, the increase in the expression of DcPSY2 gave rise to the production of carotenoids. It resulted in an increase in ABA levels of resistance in the plant.

### 4.2. High Temperature

Nascimento et al. [93] demonstrated that high temperature inhibits carrot seed germination for some carrot germplasms but not all. In the last decades, cultivar development for sub-tropical and tropical climates has advanced rapidly with the development of cultivars such as Brasilia [94,95]. This issue is becoming extremely important in several cultivation areas due to climate change. For example, the Central Valley of California, where temperate cultivars are grown, has a typical average daytime temperature of 30 °C, with a day/night range of 24–37 °C [96]. Furthermore, if adequate water is available, carrots grow widely in warm climates, such as Tunisia, Spain, and Uzbekistan. Given a scenario for even a +4 °C increase in global temperatures (GISGeography, https://gisgeography.com/climate-c, accessed on 11 December 2018), a relatively minimal threat to carrot production might be expected for most global areas if adequate water is available, based upon the success of carrot production in the Central Valley of California today.

### 4.3. Drought Stress

The effects of drought stress on carrots have been scarcely documented in scientific literature. However, with the expected reduced water availability, production would likely be severely limited in most global regions without irrigation [97], although the drought response has been reported to vary widely across diverse cultivars [98]. Reduced water availability for agriculture is expected to be especially acute in Central Asia, the Middle East, North and South Africa, and the western US [99].

## 5. Post-Harvest Physiology

### 5.1. Carrots as a Perishable Food

Carrot is one of the 10 most produced crops worldwide. In 2022, the global production of carrots and turnips was over 42 million metric tons (FAOSTAT, 2024), with China being the main producer and Europe accounting for 18.8% of world production. In the last years, fresh-cut vegetables have shown an increasing trend, which has been particularly evident during and after the recent COVID-19 pandemic [100,101]. The reasons for this positive trend are the healthy image of the product, its ease of use, and the longer shelf life compared to unprocessed vegetables; however, there are also increasing concerns for the environmental impact of fresh-cut production, as well as for the human health risks deriving from the exposure to disinfection by-products that can be found in these vegetables [102]. In any case, processed carrot perishable products, namely, baby carrots, fresh-cut carrots, and vacuum-cooked carrots, are expanding their market volumes in Western countries, also as an effect of marketing initiatives, such as Eat ‘Em Like Junk Food in the US in 2010 [103].

On the marketing side, the color of fresh-cut carrots is the primary sensory factor for consumers’ acceptance. In fresh-cut carrots, the main color degradation processes that occur are whitening and browning. Enzymatic oxidation of polyphenolic compounds relates to the browning of carrots [104], and in UV-C-treated products, its occurrence is due to the higher peroxidase (POD) activity [105]. The whitening mechanism relates to the first rescindable physical phase of dehydration and, lately, to an irretrievable physiological response linked to activation of phenolic metabolism and production of lignin [106]. Whitening index (WI) changes do not seem to affect the visual quality of carrots [107].

**Figure 8:**
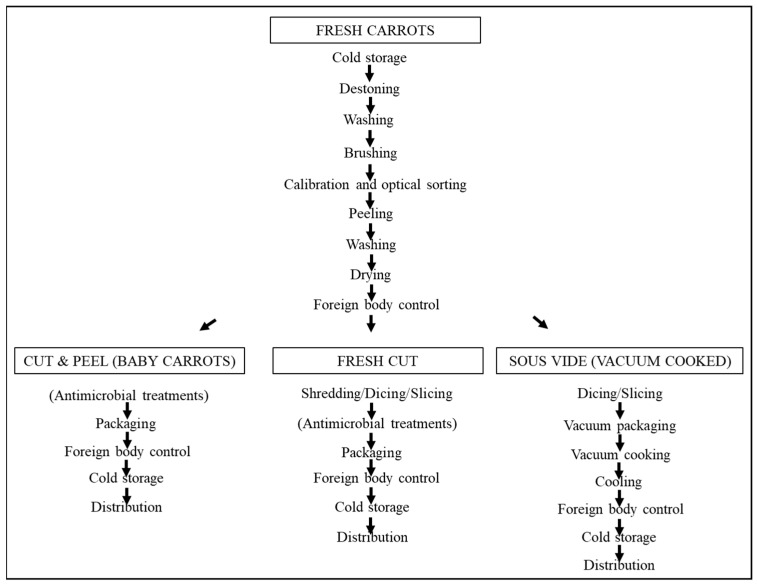
Representative patterns of perishable carrot products.

The preliminary processing steps of carrots, from acceptance of raw materials to the first foreign body control, are common. Then, in uncooked chilled carrots, antimicrobial treatments are an option that many producers consider to ensure product safety and extend the shelf life (Figure 8).

The feasibility of this option depends on regulatory constraints, producer policies, as well as on the agreements between manufacturers and distributors. In the European Union, if these antimicrobial treatments meet the requirements indicated in Regulation 1333/2008/EC Art. 3, they are considered technological aids and may be omitted on the label. On the other hand, antimicrobial treatments are not needed in vacuum-cooked carrots (or sous vide carrots), which are cooked in their own packaging and then cooled and stored under refrigeration.

As for the packaging used in perishable carrot products, vacuum is the only possible option for vacuum-cooked carrots, while the modified atmosphere is usually applied to baby carrots and fresh-cut carrots. All these products are commonly available in supermarkets, but sometimes they can also be found in small food stalls, where the important requirement of chilled storage can be critical.

### 5.2. Shelf-Life Extension

The main aspects of quality loss during post-harvest storage must be counteracted to extend the shelf life. First, lowering the respiration rate results in a longer shelf life; thus, refrigeration temperatures, modified atmospheres, and carrot coating have been the main strategies applied. Other methods, such as gaseous chlorine, ozone, and other physical technologies, have also been exploited in the later decades. These methods are mainly applied to minimally processed carrots, as washing, cutting, and slicing can stress the vegetable, increasing the exposure to spoiling microorganisms (Table 1).

Gas modification inside packaging and refrigeration can be applied to extend carrots’ shelf life. Modified atmosphere packaging (MAP) generally relies on low oxygen and high carbon dioxide percentages. The effect of different storage atmospheres was studied on chopped carrots previously sanitized in 200 mg/L of free chlorine and stored at 1 °C. Vitamin C and the approximate composition did not change in the air, under vacuum, or in MAP (2% O_2_, 10% CO_2_, and 88% N_2_), while *β*-carotene content decreased during storage, particularly in MAP. Microbial counts were low until the end of storage, independently of the atmosphere used, with psychotropic bacteria reaching the maximum value of 1.5 × 10^3^ Log CFU/g after 21 days of storage in the air [108]. Similarly, a gas atmosphere composed of 5% O_2_, 10% CO_2_, and 85% N_2_ inhibited yeast and mold growth during 21 days of storage at 4 °C, although it could not completely inhibit the development of mesophilic aerobic bacteria [109].

As already mentioned, one of the main defects of peeled carrots is the white discoloration caused by surface dehydration and lignification. To counteract this phenomenon, hygroscopic coatings made of salt solutions and polyhydric alcohols have been explored, with good results. In fact, sorbitol, glycerol, calcium chloride, calcium lactate, and propylene glycol were useful in maintaining moisture on the vegetable surface by means of a transparent layer [110]. Also, coatings based on casein, cellulose, or chitosan are effective in creating a semi-permeable barrier to oxygen and carbon dioxide, preventing moisture loss and having a preservative effect that is similar to a modified atmosphere [111]. The most suitable biopolymer is chosen based on vegetable physiology. It has the effect of slowing down the respiration rate, dehydration, gas exchange, and oxidative events, generally reducing the growth of microbial targets, thus extending the shelf life by several days and preserving qualitative and sensory attributes. Moreover, edible coatings can also be useful as carriers of anti-browning agents or antimicrobials, helping in shelf-life extension and vegetable safety improvement.

**Table 1:** Effects of treatments on carrots’ shelf-life extension and on the product microbiota and characteristics.

Treatment	Effect on Microorganisms	Effects on Carrots’ Chemical and Physical Parameters	Reference
Modified atmosphere packaging (MAP)	Growth control of the psychotropic population, inhibition of yeast and molds	Vitamin C preservation, a slight reduction in *β*-carotene, and minerals’ content decreases during storage. Negative effect on texture, preservation of color, and quality indexes.	[108,109]
Dipping/coatings based on natural polymers (alginate, casein, chitosan, etc.)	Growth control of specific spoilage organisms, *Enterobacteriaceae* and *Pseudomonadaceae*	Reduction in flavonoids and phenolic acids’ accumulation, bitterness reduction, moisture loss prevention, the anti-browning effect, color retention, and differences in antioxidant potential depending on the treatment.	[111,112]
Coatings + MAP	Load reduction and growth control of yeast and molds, coliforms, and *Pseudomonas* spp.	Moisture loss prevention, respiration increase, prolonged firmness, prevention of surface whitening, color and texture retention.	[113]
Ozonation/ozonated water	Inhibition of *Escherichia coli* O157:H7, STEC *E. coli*, *Salmonella enterica*, *Listeria monocytogenes*, and *Pectobacterium carotovorum.* Fungistatic effect on *B. cinerea* and *S. sclerotiorum*	Delay of carrot thickening, maintenance of pH, dose-dependent oxidative damages: pigment disruption, color change, increased respiratory rate, dehydration, and electrolyte loss.	[114,115,116,117,118]
Ozone + UV-C rays	Reduction of total mesophilic population and coliforms. No effect on yeast and molds.	Not reported.	[119]
Ozone + MAP	Inhibition of microorganisms on the product surface. Reduction of total mesophilic population.	Reduction in total phenolics, enzyme activity, respiration, and ethylene rate, retention of total carotenoids and ascorbic acid, color maintenance.	[116]
Chlorine dioxide	Reduction of mesophilic and psychrotrophic population, including lactic acid bacteria. Scarce effect on yeast that determined the shelf life.	Moisture loss prevention, white discoloration prevention, slight pH reduction, and maintenance of sensory attributes.	[120]
High pressure	Inactivation of vegetative cells.	Maintenance of texture, red color, and carotenoid content, as well as dry matter reduction. Increase of free and bound phenolics, increase of antioxidant content.	[121,122]
UV-C treatment	Variable inhibition of microbial growth, depending on the wavelength. Reduction in *Sclerotinia sclerotorium* load.	Maintenance of aroma, color, nutritional, and physical–chemical characteristics.	[123,124]
Gamma irradiation	Limited effect because of the legal restrictions in the doses applicable.	Maintenance of quality attributes.	[125]
Irradiation + active coating	Reduction of total mesophilic population and yeast and mold count.	Improvement of mechanical and water vapor barrier characteristics of the coating, maintenance of weight, firmness, and color.	[125]
Nisin + plant extracts + irradiation	Reduction of total mesophilic population, yeast and molds, and *Listeria monocytogenes* count.	Maintenance of weight, firmness, and color.	[126]
Different essential oils	Reduction of *Sclerotinia sclerotiorum* growth.	Increase in enzymes (polyphenol oxidase, peroxidases, chitinases, etc.) content, inducers of resistance against the molds.	[127]
*Coriandrum sativum* EO	Reduction of *Salmonella enterica* growth.	Maintenance of sensory traits of the product, as well as color stability.	[76]
Thyme EO	Reduction of *Escherichia coli* O157:H7 count.	Not reported.	[115]
Thyme EO + ClO_2_ + ozonated water	Effective reduction of *Escherichia coli* O157:H7 count.	Not reported.	[115]
Microencapsulated chitosan + thyme EO	Reduction and control of mesophilic, psychrophilic, yeast, and mold populations during time.	Increase of total phenolics content (TPC) and antioxidant capacity.	[128]

In addition, dipping (i.e., in ethanol) and application of edible coatings (i.e., alginate) can control the product dehydration and the microbial development, particularly of specific spoilage bacteria, such as *Enterobacteriaceae* and *Pseudomonas* spp., still preserving sensory properties, with a significant shelf-life extension up to 12–13 days [112]. Furthermore, edible coatings have the advantage of being produced from fruit and vegetable by-products and residues, consequently improving the quality of the treated vegetables and converting waste into a functional film with added value. Moreover, functional coatings can be suitable for spraying and dipping, depending on the needs of the final product. In general, a larger vegetable surface exposed to the coating determines a more efficient protective effect of the layer, as the tissue absorbs part of the coating. Therefore, shredded carrots yield better results than sliced ones [129]. Chitosan powder has been directly applied to carrot shreds at concentrations up to 0.4% and stored in LDPE bags at 10 °C for 10 days. The treated samples had mesophilic loads lower than 1.3 log CFU/g with respect to the control, and the treatment also significantly reduced yeasts and molds, determining minimal pH, titrable acidity, and total solid content variations. The microbiological and sensory quality of treated samples was superior after 10 days of storage, while controls were acceptable only up to 5 days of storage [130].

Different preservation methods can also be combined, such as chitosan-based coatings, MAP, and refrigerated storage of baby carrots, with a better effect on microbial spoilage delay with respect to individually applied strategies. Chitosan alone already exerts antimicrobial activity, and the combination of all the hurdles can help control the growth of total viable microorganisms, yeasts and molds, coliforms, and *Pseudomonas* spp. over time [113].

Recently, different physical methods have been proposed for carrot treatment to extend the products’ shelf life and/or improve their safety; for example, ozone, to be applied as a gas or as ozonated water [114]. Different effectiveness has been reported, depending on the duration of the treatment, ozone concentration, and the microbial target. For example, increasing the storage temperature would require more ozone to guarantee a specific residual concentration. There appears to exist a threshold in ozone concentration (up to 5 mg/L of gaseous ozone and up to 10 mg/L of ozone dissolved in water), above which the exposure can cause damage to the vegetable. Below these doses, ozone delays carrots’ thickening and inhibits microorganisms, extending carrots’ shelf life, although ozone in water can temporarily affect the internal pH [114]. The antimicrobial effect on *E. coli* O157:H7, STEC *E. coli*, *Salmonella enterica*, and *Listeria monocytogenes* has been proven and seems to increase with concentrations and time of exposure [115,116]. According to Hassenberg et al. [117], ozonized water at a concentration of 4 ppm for 2 min inhibited *Pectobacterium carotovorum* in washed carrots, without leaving any hazardous residue. Nevertheless, according to other authors, the effect on fungi, such as *B. cinerea* and *S. sclerotiorum*, is only fungistatic and not fungicidal. At the same time, concentrations comprised between 10 and 22 µL/L at 2 °C, because of their oxidative effect, caused physiological damages to the vegetable, including color change due to pigment destruction, and increased the respiration rate and loss of electrolytes [118]. Due to the oxidation power of ozone, terpenes can also increase in the headspace.

Chlorine dioxide (ClO_2_) has been studied for the shelf-life extension of minimally processed carrots. Unlike liquid chlorine and hypochlorite, chlorine dioxide does not react with ammonia-forming chloramines, which are toxic for workers and consumers, but still behaves as a strong oxidizing agent with an antimicrobial effect on surfaces. This gas can inhibit microbial growth, keep carrot tissues hydrated, and thus reduce the risk of white blush discoloration [120]. A treatment of 6 min at 28 °C with a maximum ClO_2_ concentration of 1.33 mg/L significantly reduced mesophilic and psychotropic bacteria, particularly lactic acid bacteria, preserving the sensory attributes of carrot sticks. Unfortunately, the treatment was less effective on yeasts, which allowed a shelf-life extension of only one day, reaching a load greater than 5 Log CFU/g after five days of storage [120].

During the last decades, high-pressure processing (HPP) has emerged as a non-thermal process in which food products are subjected to a pressure of 400–600 MPa at room or refrigerated temperature for a variable time of a few minutes [121]. The treatment inactivates vegetative microbial cells, extending the products’ shelf life. Specifically, in carrots, HPP treatments guarantee better texture preservation and red color retention than thermal treatments, considering treatments with an equivalent effect on microbial inactivation [121]. Moreover, HPP processing of whole carrots at mild conditions (60–100 MPa for 5 min) can increase the content of natural antioxidants, such as free and bound phenolic compounds, preserving the carotenoid content [122].

Among the physical methods, UV-C treatment is a non-thermal disinfection method mainly used for surfaces. The strongest antimicrobial effect is reached when radiation at 253.7 nm is applied. The radiation hits the microbial DNA, preventing its transcription and translation and, therefore, inhibiting microbial growth. The treatment generally maintains the qualitative, nutritional, and physical–chemical characteristics of the product, without affecting aroma and color [123]. The application of UV-C radiation with a peak at 254 nm for 5 min significantly decreased the *S. sclerotorium* load [124]. Nevertheless, some authors applied UV-C at 253 nm on carrots and obtained a microbial reduction of about 1 Log CFU/mL, which was insufficient for shelf-life extension [123].

Gamma irradiation is another physical, non-thermal method to assure food safety, which can be applied to fresh vegetable products. Although considered safe for consumers by the Codex Alimentarius Commission, World Health Organization, FAO, and International Atomic Energy Agency, food irradiation is not allowed in all nations. Still, it has been approved in over 60 countries [131]. Nonetheless, the doses necessary to inhibit pathogenic microorganisms to an undetectable level usually exceed the dose recommended for fruit and vegetables, which is below 1 kGy, although the nutritional quality of this product is preserved at irradiation doses up to 10 kGy [125]. Consequently, the approach based on the “hurdle technology” is frequently applied to overcome the limited antimicrobial effectiveness of these physical methods. In detail, combining different preservation methods or techniques can improve food safety and extend the shelf life without reducing the nutritional and sensory quality of the product. For example, the combination of ozone and UV-C rays [119], ozone with modified atmosphere [116], or even irradiation and bioactive coating based on calcium caseinate incorporated with citrus extract, cranberry juice, and essential oils [125], and nisin plus carvacrol or mountain savory and then irradiation at 0.5 and 1.0 KGy [126], have been proposed. The combination of different hurdles and/or technologies generally shows a synergistic potential and a higher efficiency in extending carrots’ shelf life, compared with the same treatments singularly applied.

Finally, essential oils and plant extracts have also been applied to counteract microbial pathogens’ growth or extend the shelf life of carrots. The effects of different essential oils (EOs) were tested against *Sclerotinia sclerotiorum* during carrots’ storage. Thyme and savory EOs not only were the most effective but also increased the level of peroxidases, chitinases, polyphenol oxidase, and other enzymes, therefore showing the potential to induce resistance of the vegetable against the white mold [132]. In another study, Pellegrini et al. [76] applied *Coriandrum sativum* essential oil (5 μL/mL) as a washing treatment on carrot sticks. Two minutes of contact reduced the load of a cocktail of three *Salmonella enterica* strains of about 1 Log CFU/g for up to 24 h, without affecting the sensory profile of the product. The authors suggested applying this washing treatment in combination with other treatments to boost this effect. While washing baby carrots for 5 min with thyme essential oil (1.0 mL/L) determined more than a 1.0 Log CFU/g reduction of inoculated *Escherichia coli* O157:H7, the sequential washing by means of thyme EO, ClO_2_, and ozonated water was significantly more effective, reducing the *E. coli* load by more than 3.75 Log CFU/g [115]. A chitosan coating containing free thyme EO and microencapsulated in *β*-cyclodextrin was demonstrated to exert antimicrobial activity on mesophilic, psychrophilic, yeasts, and mold populations and to extend the shelf life of sliced carrots. While the first combination immediately reduced the microbial count, the effect was lost during storage. The coating containing microencapsulated thyme EO reduced the load from day 6 until the end of storage [128].

As a result of all the studies previously described, a combination of different techniques is the most effective strategy to extend carrots’ shelf life from microbiological, nutritional, sensory, and technological points of view.

## 6. Carrot Breeding: Genetic Resources and Genomic Selection

Carrot accessions in the germplasm are the treasured sources of desired traits with genetic diversity. The genotyping of total accessions in germplasm collections makes it likely to use genomic prediction for valued accession identification and exclude less desired trait values [133]. Genomic prediction of accessions by different strategies offers a convincing landscape of breeding programs over field screening. The introgression of desired traits as accessions into stand-out breeding lines by means of genomic and phenotypic selection leads to new genetic makeup for improving carrot varieties.

*D. carota* is a cross-pollinated diploid species. It is vital in human nutrition and agro-economy [134]. Carrots, canopy height, and flavor are quantitative traits with moderate heritability. In breeding for weeds, lowness vs. height of the canopy is a goal in the selection process [135,136]. Several carrot accessions have harsh flavors due to volatile terpenoids, but selected varieties typically have mild (non-harsh) features. Upholding mild flavor while breeding canopy height traits into elite breeding lines is now an engrossed breeding goal [43].

In fact, the engrossed breeding goal for carrots is to breed varieties with tall canopies and mild flavor as a model vegetable crop. In breeding contexts, genomic prediction strategies can assist in identifying valuable breeding material with high-density genotype data to predict phenotypes or breeding values in collections/germplasms. The application of genomic prediction requires designated populations to be estimated in a potential breeding context. The genomic selection will possibly allow the identification of valuable accessions without requiring extensive field evaluation. Therefore, it appears to yield similar results as phenotypic selection, with lower costs for phenotyping. Thus, assessing a training population in the target environment may be strategic for some traits [137].

Corak et al. [137] compared the performance of two genomic selection strategies. The first method, the genomic-breeding population (GBP), uses past data of phenotypes to forecast the accession with a genetic value of additives so that field screening of accessions in selecting parents is avoided. The second method, the genomic-training population (GTP) method, employs data from a training population of representative phenotypes in an environment of the target [138]. These methods have limits to assessing accessions in the field, with strong access to genotypic data. These two genomic selection strategies with phenotype selection (PS) were tested to identify carrot accessions with a tall canopy height and good flavor. In this study, a selection model trained on phenotypes from only 10% of the collection was found to be the most promising. This means that the trade-off in prediction accuracy and the cost of phenotyping could be balanced using an optimum training population size, which is key to identifying and excluding unwanted accessions. The results obtained in [137] demonstrated that populations derived from crosses between highly ranked accessions of parental and selective inbreds showed similar trait distributions. With additional cycles in selection, the results of the GTP group selection at the F2 generation can be considered encouraging, as reported by Corak et al. [138]. Even in a larger training population, GTP would reduce the expanse of phenotyping needed prior to selecting parental accessions for target traits.

In the future, the improvement of carrots is likely to be assisted by artificial intelligence and machine learning methods, which can analyze the correlation between various attributes, such as yield and nutritional characteristics. This issue is extremely relevant for human nutrition, considering that 28–90% of total *β*-carotene for humans comes from carrots. Moreover, the data on the nutritional traits of genotypes complement essential phenotypic and genetic characterization and its association with color variation. Riaz et al. [139] reported that various morpho-nutrition traits were estimated in 64 genotypes collected from 4 continents. An evaluation of genetic variability, heritability, strength, and direction of association among variables, and direct and indirect relationships among physicochemical and nutritional traits with *β*-carotene content was assessed. A significant association with *β*-carotene accumulation was noted with core diameter, foliage weight, root weight, and shoulder weight. Principal component analysis divided genotypes into two typical groups: Eastern and Western carrots. It was revealed that caloric and moisture content had high positive links with *β*-carotene content, while carbohydrate content was negatively associated. In this study, five genotypes (T-29, PI 634658, PI 288765, PI 164798, and Ames 25043) with the highest *β*-carotene contents were selected and used for making three nutraceutical supplements (carrot–orange juice, carrot jam, and carrot candies). These nutraceutical supplements retained a high *β*-carotene content coupled with antioxidant properties [139].

Koutouan et al. [44] screened a total of 300 accessions from carrot genetic resources in Angers (France) and other European genetic resources from 1997 to 2000 for their resistance to *Alternaria dauci* in different environments. Based on the authors’ findings, three inbred lines, namely K3, I2, and Boléro, were highly resistant, whereas genotype H1 was highly susceptible. The selection was based on several quantitative trait loci (QTL) associated with resistance to *A. dauci.* The different genotypes showed varied resistance mechanisms for the QTLs involved [140,141]. The strategy described by Koutouan et al. [44] can be useful to identify the genotype with the highest resistance.

## 7. Future Prospects

For carrot seed companies, one of the core breeding objectives is to increase the resistance level of new cultivars in one genotype by accumulating complementary resistance factors while breeding for less weeds. Canopy height and flavor are the two quantitative traits that uphold the mild flavor. In contrast, breeding canopy height into elite breeding lines is now an engrossed breeding goal.

Although carrots are a well-known and explored staple food, increasing efforts to improve their safety and shelf life have been documented in recent literature. In this respect, also in view of the challenges generated by climate change, future strategies for post-harvest storage and processing are likely to be based on a combination of methods. Finally, in the framework of circular economy, carrots will become a source of important bioactive compounds and by-products, which will be explored in different industrial environments. In this respect, the data gathered in this review can be considered a valuable toolbox for both crop scientists and food technologists.

## Data Availability

Not applicable.
